# BODY MASS INDEX AND ALBUMIN LEVELS ARE ASSOCIATED WITH PULMONARY FUNCTION PARAMETERS IN PEDIATRIC SUBJECTS WITH CYSTIC FIBROSIS

**DOI:** 10.1590/1984-0462/;2019;37;4;00016

**Published:** 2019-05-09

**Authors:** Miriam Isabel Souza dos Santos Simon, Gabriele Carra Forte Paulo, José Cauduro Marostica

**Affiliations:** a Universidade Federal do Rio Grande do Sul, Porto Alegre, RS, Brazil.

**Keywords:** Cystic fibrosis, Pediatrics, Nutritional assessment, Albumins, Body mass index, Fibrose cística, Pediatria, Avaliação nutricional, Albuminas, Índice de massa corporal

## Abstract

**Objective:**

To evaluate the association of body mass index (BMI) and albumin with pulmonary function in cystic fibrosis (CF) pediatric subjects.

**Methods:**

This is a cross-sectional study with clinically stable CF’s subjects. Clinical (pulmonary function) and nutritional evaluation (body mass index and albumin) were performed. Univariate analysis was performed using simple linear correlations. Regression analysis was performed using an exit level of p<0.05.

**Results:**

Seventy-eight CF’s subjects (mean age 12.8±3.8 years) with mean albumin 4.2±0.4 mg/dL, predicted forced expiratory volume in 1 second (FEV1%) 80.8±22.6 and BMI median percentile 51.2 (1.3-97.7). In the multiple regression models, albumin, age and BMI percentile were associated with pulmonary function. Subjects with lower than 25 BMI percentile had 12.2% lower FEV_1_%. An albumin increase of 0.1 mg was associated with 2.7% increase in predicted FEV1%, and one year increase in age was associated with reduction in 1.2% of predicted FEV1%.

**Conclusions:**

BMI percentile, albumin and age were independently associated with predicted FEV1% in a tertiary referral hospital.

## RESUMO

**Objetivo:**

Avaliar a associação do Índice de Massa Corporal (IMC) e da albumina com a função pulmonar em pacientes pediátricos com fibrose cística (FC).

**Métodos:**

Estudo transversal com pacientes pediátricos com FC clinicamente estáveis. Foram realizadas avaliação clínica (função pulmonar) e nutricional (IMC e albumina). Análise univariada foi realizada usando correlação linear simples. Análise de regressão foi realizada usando o nível de significância de p<0,05.

**Resultados:**

Foram incluídos 78 pacientes com FC (média de idade 12,8±3,8 anos) com média de albumina de 4,2±0,4 mg/dL, volume expiratório forçado em um segundo (VEF_1_%) predito de 80,8±22,6 e mediana do percentual de IMC de 51,2 (1,3-97,7). No modelo de regressão múltipla, albumina, idade e percentual de IMC apresentaram associação com a função pulmonar. Indivíduos com IMC abaixo de 25% apresentaram VEF_1_% predito 12,2% menor. Um aumento de 0,1 mg de albumina teve associação com aumento de 2,7% no VEF_1_% predito, e um ano a mais de idade mostrou relação com a redução de 1,2% de VEF_1_% predito.

**Conclusão:**

O percentual de IMC, albumina e idade apresentaram associação independente com o VEF_1_% predito em um hospital terciário de referência.

## INTRODUCTION

Cystic fibrosis (CF) lung disease is associated with morbidity, and pulmonary function is the most important predictor of survival. Forced expiratory volume in 1 second (FEV_1_%) is regarded as the best generally available measure for assessing CF lung disease.^1^ Various factors are potentially associated with FEV_1_%, such as nutritional status, chronic airway infection and oxidative stress.^2^

Association of lung function with nutritional status has long been recognized. Several studies have shown that weight gain leads to an improved pulmonary function, whereas weight loss can accelerate pulmonary function decline.^2^ ^,^ ^3^ ^,^ ^4^ ^,^ ^5^ Stallings et al.^6^ showed that better FEV_1_% status at about 80% predicted or above was associated with body mass index (BMI) percentiles at the 50th percentile and higher. A previous study demonstrated that weigh-for-age ≥10th percentile at age were associated with higher survival at 18 years.^7^

Presence of chronic *Pseudomonas aeruginosa* in respiratory tract has been previously reported to be associated with faster decline in lung function in CF subjects. Studies showed a significantly association of chronic *Pseudomonas aeruginosa* infection with lower FEV_1_%.^3^ ^,^ ^8^ ^,^ ^9^ In addition, such infection is well recognized as a predictor of morbidity and mortality in young children with CF.^10^ ^,^ ^11^

The pathophysiology of CF is one of increased inflammation-related oxidative stress, particularly during exacerbations.^12^ Lungs have multiple layers of defense against persistent oxidant stress, and albumin is an important non-enzymatic antioxidant of interest in this disease. In addition, it is the most abundant multifunctional plasma protein and it has been noted to be a substantial contributor to systemic antioxidant capability, as well as having anti-inflammatory functions.^13^

In a previous study, Khatri et al.^13^ observed that albumin was significantly correlated with lung function and asthma quality of life scores. There is only one study that demonstrated albumin as a predictor of lung function in CF subjects.^14^

The objective of this study was to evaluate the association of BMI and albumin with pulmonary function in CF pediatric subjects.

## METHOD

This was a cross-sectional study. Subjects were consecutively recruited from the pediatric CF outpatient clinic at the Hospital de Clínicas de Porto Alegre, Rio Grande do Sul, Brazil. Subjects were eligible if they had a confirmed CF diagnosis by at least two abnormal sweat chloride test results. Subjects experiencing a pulmonary exacerbation in the last two weeks were excluded. The study was approved by the Human Research Ethics Committee (Protocol no. 09-429). Demographic, clinical, nutritional and biochemical data were obtained from patient medical records.

All participants underwent a clinical and nutritional evaluation. Clinical and demographic data such as age, age at diagnosis, pancreatic insufficiency (denoted by use of enzyme replacement therapy), genetic mutation analysis, presence or absence of bacterial colonization (*Staphylococcus aureus*, methicillin-resistant *Staphylococcus aureus -* MRSA, *Pseudomonas aeruginosa*, mucoid *Pseudomonas aeruginosa* and *Burkholderia cepacia complex*), plasma albumin levels, and FEV_1_% predicted were obtained from subject charts, and all the variables were acquired at the moment of the patient check-ups.

A flow-volume curve was performed in the Master Screen Jaeger^®^ spirometer (Würzburg, Germany), using the Zapletal table for the expected values. Spirometry was always performed by the same examiner and its quality was checked by the attending physician by analyzing the curves. Spirometry was done according to the guidelines for Pulmonary Function testing 2002.^15^ FEV_1_% was chosen for analysis, since it is the most widely used parameter in the literature to quantify the obstructive ventilatory damage characteristic of CF.

Nutrition data (weight and height measurement) were analyzed as percentiles according to the World Health Organization (WHO) equations (weight/age; height/age; BMI/age). The cut-off point established to assess the relationship between nutritional status and lung function was the 25th percentile, according to the study of Konstan et al.^16^

Sample size was calculated considering r=0.42 in the association between FEV_1_% and albumin, according to previous data from Simon et al.^14^ Seventy-five cases were estimated based on the 5% level of significance, 95% confidence interval, and statistical power of 95%.

Descriptive statistics were used to describe subject characteristics. We first constructed regression models for FEV_1_% as the main outcome, using anthropometric, biochemical and clinical data as independent variables. Univariate analysis was performed using simple linear correlations. Where a significant relationship was identified (p<0.10), a stepwise multiple linear regression was done using FEV_1_% as the dependent variable and significantly associated anthropometric and biochemical data as the independent variables, controlling for age and sex. Regression analysis was performed using the exit level of p<0.05. All analyses were done using Statistical Package for the Social Sciences (SPSS) for Windows version 18.0 (IBM, Armonk, NY, United States).

## RESULTS

Seventy-eight CF pediatric subjects participated in the study; 50% were female. The mean age of participants was 12.8±3.8 years old, the median of age at diagnosis was 2.1 years old. Mean albumin was 4.2±0.4 mg/dL, and FEV_1_% predicted was 80.8±22.6, the BMI median percentile was 51.2 ­(1.3-97.7). Additional clinical data and other characteristics are provided in Table 1. The results of the multiple linear regression analyses for FEV_1_% as the main outcome are shown in Table 2.

**Table 1:** Demographic and clinical characteristics of the children and adolescents patients with cystic fibrosis.

	n=78
Gender, n (%)	
Females	39 (50)
Age (years), mean±SD	12.8±3.8
Age at diagnosis (years), median (IR)	1.3 (0.4-6.0)
Mutation, n (%)	
∆508 Homozygosis	29 (37.2)
∆508 Heterozygosis	19 (24.4)
Others	8 (10.3)
Pancreatic insufficiency, n (%)	69 (88.5)
Albumin, mean±SD	4.2±0.4
Bacterial colonization, n (%)	
*Staphylococcus aureus*	60 (76.9)
Methicillin resistant *Staphylococcus aureus*	9 (11.5)
*Pseudomonas aeruginosa*	42 (53.8)
*Pseudomonas aeruginosa* mucoid	15 (19.2)
*Burkholderia cepacia*	19 (24.4)
Pulmonary function, mean±SD	
FEV_1_% predicted	81.9±22.6
Nutritional markers, mean±SD	
Weight, percentile	52.6±.26.3
Height, percentile	43.3±26.9
BMI, percentile	49.9±27

n: number of cases; SD: standard deviation; IR: interquartile range; FEV_1_%: forced expiratory volume in one second; BMI: body mass index.

**Table 2:** Multiple linear regression analysis for pulmonary function in cystic fibrosis patients.

Pulmonary function	Multiple regression	β (95%CI)	Adjusted R^2^
FEV_1_% predicted	BMI<25 percentile	-12.2 (-21.9 to -2.6)*	0.368
	Age (years)	-1.2 (-2.3 to -0,1)*	
	Albumin	2.7 (1.6 to 3.8)**	

*p-value<0.01; **p-value<0.001; 95%CI: 95% confidence interval; FEV_1_%: forced expiratory volume in one second; BMI: body mass index.

In the multiple regression models, albumin, age and BMI percentile were associated with pulmonary function. Subjects with lower than 25 BMI percentile had 12.2% lower FEV_1_%. An albumin increase of 0.1 mg was associated with 2.7% increase in FEV_1_% predicted, and one year increase in age was associated with reduction in 1.2% of FEV_1_% predicted.

## DISCUSSION

This study demonstrates that FEV_1_% has a direct association with BMI lower than 25th percentile, with age, and with albumin levels, underscoring the hypothesis that good nutritional status and albumin levels are important in CF. The regression model was able to explain approximately 40% of FEV1% variability.

Association of FEV_1_% with BMI percentile is well recognized.^4^ ^,^ ^6^ ^,^ ^17^ ^,^ ^18^ Our data demonstrated that being below the BMI 25th percentile was associated with 12% lower FEV_1_%. Stallings et al.^6^ showed that BMI percentiles at 25th was associated with FEV_1_% status below 90% predicted in subjects aged 6 to 12 years old, and below 80% in subjects aged 13 to 20. In our previous study, we observed that subjects with a BMI below the 10th percentile had 25.58% lower FEV_1_%.^14^ Our data show that, even in subjects who were not malnourished, reduction in pulmonary function parameters was found.

In CF, lung function decreases with time, and it is thought that even a small decline of 1-2% per year is deleterious in the life expectancy of the subjects.^19^ We observed similar results in this sample, in which one year increase in age was associated with reduction of approximately 1% of FEV_1_% predicted.

The present study data demonstrate that an albumin increase of 0.1 mg was associated with 2.72% increase in FEV_1_% predicted, even having most of the subjects a normal albumin level. In our previous study, we found that plasma albumin levels lower than or equal to 4.1 mg/dL predicted 18.6% fall in FEV_1_%.^14^ Besides that, we observed that plasma albumin levels of 4.2 mg/dL were predictive of FEV_1_% of 60% with good sensitivity, specificity and accuracy. Therefore, we hypothesized that albumin is an indicator of inflammatory process, and for this reason it is related with poor pulmonary function.^20^ Khatri et al.^13^ showed that plasma albumin levels directly correlated with FEV_1_% predicted among asthmatic subjects (R=0.378; p=0.010).

In the current study, chronic *Pseudomonas aeruginosa* infection was not significantly associated with decreased lung function. These results could be explained by chronic *Pseudomonas aeruginosa* infection and age being covariates and, for this reason, loosing statistical power. However, other studies^3^ ^,^ ^21^ showed significant association of *Pseudomonas aeruginosa* and lower lung function.

One of the limitations of our study was the cross-sectional design, so findings do not necessary reflect causality.

In conclusion, in pediatric CF’s subjects BMI percentile, albumin and age were independently associated with FEV_1_% predicted in a tertiary referral hospital. The results emphasize the relevance of evaluate the association between albumin levels and lung function in CF.
