# ERRATUM

**DOI:** 10.1590/1984-0462/;2019;37;4;00016erratum

**Published:** 2020-05-11

In the manuscript “Body mass index and albumin levels are associated with pulmonary function parameters in pediatric subjects with cystic fibrosis”, DOI: 10.1590/1984-0462/;2019;37;4;00016, published in the Rev. Paul. pediatr. 2019;37(4):414-418. Epub May 09, 2019, on page 414.

**Where it reads:**

Gabriele Carra Forte Paulo, José Cauduro Marostica

**It should read:**

Gabriele Carra Forte, Paulo José Cauduro Marostica

